# Transforming Growth Factor-Beta in Skeletal Muscle Wasting

**DOI:** 10.3390/ijms23031167

**Published:** 2022-01-21

**Authors:** Gordon L. Klein

**Affiliations:** Department of Orthopaedic Surgery, University of Texas Medical Branch, Galveston, TX 77555-0165, USA; gordonklein@ymail.com

**Keywords:** transforming growth factor-beta, skeletal muscle wasting, reactive oxygen species, metastatic cancer, pediatric burns

## Abstract

Transforming growth factor-beta (TGF-β) is part of a family of molecules that is present in many body tissues and performs many different functions. Evidence has been obtained from mice and human cancer patients with bony metastases and non-metastatic disease, as well as pediatric burn patients, that inflammation leads to bone resorption and release of TGF-β from the bone matrix with paracrine effects on muscle protein balance, possibly mediated by the generation of reactive oxygen species. Whether immobilization, which confounds the etiology of bone resorption in burn injury, also leads to the release of TGF-β from bone contributing to muscle wasting in other conditions is unclear. The use of anti-resorptive therapy in both metastatic cancer patients and pediatric burn patients has been successful in the prevention of muscle wasting, thereby creating an additional therapeutic niche for this class of drugs. The liberation of TGF-β may be one way in which bone helps to control muscle mass, but further investigation will be necessary to assess whether the rate of bone resorption is the determining factor for the release of TGF-β. Moreover, whether different resorptive conditions, such as immobilization and hyperparathyroidism, also involve TGF-β release in the pathogenesis of muscle wasting needs to be investigated.

## 1. Introduction and Early Work

This review will discuss the influence of bone resorption on muscle wasting by liberation of transforming growth factor-beta from the matrix of resorbing bone and apparent paracrine catabolic effects on skeletal muscle.

Transforming growth factor (TGF)-β is the name for a superfamily of proteins, including myostatin, that functions in the body to affect growth and to stimulate the inflammatory response along with other functions covered elsewhere. For our purposes, the function of fibrogenesis is of particular importance because it was the main stimulus for the initial investigations of the effects of TGF-β on skeletal muscle [1]. 

The findings from several studies correlated the quantity of TGF-β in skeletal muscle with the amount of fibrosis detected in muscle tissue in mouse models of Duchenne Muscular Dystrophy (DMD; [2,3,4]). They also established a relationship between muscle damage at the onset of DMD pathology or of experimental muscle injury and marked increases in skeletal muscle TGF-β accumulation, as well as the fibroadipogenesis and fibrocalcification of muscle [5] in these mouse models of DMD. Gonzalez et al. [6] showed that muscle from a mouse model of amyotrophic lateral sclerosis (ALS), a fatal neurodegenerative disease, expressed increased amounts of TGF-β and Smad3, increased fibrosis, and induction of fibroadipogenic precursors. These studies collectively indicate that muscle damage may result in increased TGF-β deposition in muscle, and that neuromuscular degenerative diseases may also have TGF-β signaling involved in their pathogenesis. Therefore, it is possible that TGF-β accumulation in skeletal muscle can be either a primary or a secondary event contributing to the pathogenesis of muscle fibrosis in DMD as well as ALS. 

Work by Abrigo et al. [7] indicated that TGF-β in skeletal muscle not only contributed to muscle fibrosis in DMD disease, but also promoted skeletal muscle atrophy by decreasing muscle fiber diameter and amounts of heavy chain myosin (MHC) in muscle tissue. Additionally, these investigators found increasing amounts of the E3 ubiquitin ligase MuRF-1 expression, indicating TGF-β stimulation of the catabolic ubiquitin ligase pathway. Moreover, they reported that an upstream event from the TGF-β stimulation of either the canonical SMAD 2/3 signaling pathway or the non-canonical ERK 1/2 and JNK 1/2 signaling pathways was the stimulation of reactive oxygen species (ROS) production, possibly initiated by angiotensin II [7]. TGF-β stimulation of the SMAD and ERK and JNK signaling pathways can be blocked by the angiotensin II inhibitor angiotensin (1–7) [8]. It is not known whether angiotensin II stimulates ROS generation as a direct consequence of the presence of TGF-β in skeletal muscle, especially since tissues such as kidney can demonstrate resistance in the form of vascular tachyphylaxis to the effects of angiotensin II [9]. The circumstances surrounding the role of angiotensin II in the pathogenesis of reactive oxygen species requires further investigation. Thus, TGF-β has been implicated not only in the pathogenesis of skeletal muscle fibrosis in genetic diseases such as DMD, but also in skeletal muscle wasting. While during the first decade of the 21st century there was no evidence for the clinical importance of TGF-β stimulation of muscle wasting in disease states, what was also missing was evidence for the origin of the TGF-β that would be found in muscle inasmuch as the initial studies involved direct administration of TGF-β to muscle [7,8].

## 2. The Role of Bone

**Figure 1:**
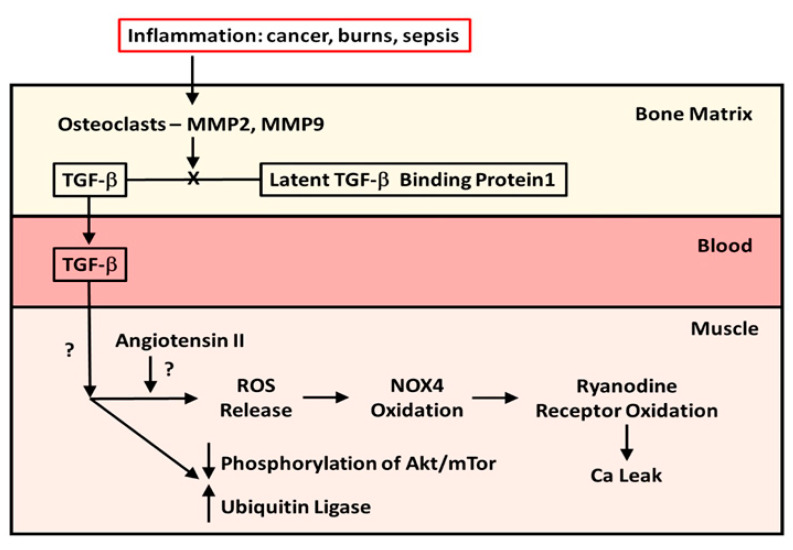
The mechanism and effect of release of TGF-β from bone matrix. Diagram of the cleavage of TGF-β from Latent TGF-β Binding Protein-1 by osteoclasts in the bone matrix during resorption and the effects of TGF-β released from bone during resorption on muscle protein metabolism in patients with cancer metastatic to bone and in pediatric burn patients.

In 2015, Waning et al. [10] described changes to the bone microenvironment in both mouse models and in patients with bone metastases from breast and lung cancer. The work of this group identified that bony metastases from cancers resulted in inflammation, with resultant liberation of TGF-β from bone. The result of this release was the generation of ROS in the sarcoplasmic reticulum of skeletal muscle, where it interacts with the ryanodine receptor, oxidizing it and causing a calcium leak resulting in muscle weakness. The effects of TGF-β on the ryanodine receptor could be reversed by use of either a TGF-β1 receptor blocker or the anti-resorptive agent zoledronate. This study confirmed the work of Abrigo et al. [7] in identifying ROS generation as a mode of action leading to muscle weakness in metastatic cancer. Another piece of the puzzle, the mechanics of the release of TGF-β from resorbing bone, was supplied by the work of Dallas et al. [11]. This group reported that TGF-β was stored in the bone extracellular matrix bound to Latent TGF-β Binding Protein-1 and that osteoclasts, with the aid of matrix metalloproteinases (MMP)-2 and -9, can cleave this binding protein from TGF-β, liberating it from the extracellular matrix during resorption. Figure 1 illustrates the known pathway of TGF-β release from bone. What was, at the time, unclear was whether this TGF-β liberation from bone with paracrine catabolic effects on muscle was unique to metastatic cancers. As it turns out, Pin et al. [12] subsequently described muscle wasting in cancer without metastases, inasmuch as IL-6, tumor necrosis factor-alpha (TNF-α), interferon-gamma (IFN-¥) and IL-1-β are chronically elevated in blood, peripheral tissues, and the central nervous system of patients with cancer cachexia. These cytokines can be produced by either the immune cells or tumor cells. Moreover, myostatin, a member of the TGF-β family of proteins, and one that inhibits muscle protein synthesis, has a catabolic effect on bone by inhibiting osteoblast differentiation by decreasing the osteocyte-derived production of exosomal microRNA (mIR) 218 [13]. Myostatin also increases RANK Ligand activity in stimulating osteoclast formation. To compound matters, Chen et al. [14] showed that the inhibition of activin receptors and of myostatin inhibited the Smad 2/3 pathway, producing muscle hypertrophy. The driving factor in the muscle hypertrophy was an increase in muscle protein synthesis and a reduction in ubiquitin ligase activity—findings seen with the inhibition of TGF-β release from bone. Thus, the relationship between the TGF-β molecule liberated from bone during resorption, and muscle myostatin activity, remains unclear.

However, it is known that both TGF-β1 and myostatin bind to the same receptors, which, when bound, will phosphorylate the Smad 2/3 signaling pathway, resulting in stimulation of the ubiquitin ligase muscle protein catabolic pathway and suppression of the Akt/mTor muscle protein anabolic pathway. 

Not only is muscle wasting produced by cancer metastases and cancer without metastases, but chemotherapeutic agents, such as carboplatin [15] and cisplatin [16], are capable of producing bone loss and consequent muscle wasting with zoledronate preventing the muscle wasting in both instances. The authors of these papers did not specify that activation of TGF-β was involved in the pathogenesis of the muscle wasting.

### Expansion of the Role of Bone-Liberated TGF-β to Other Musculoskeletal Conditions

At about the same time as Waning et al. [10] were carrying out their studies, our group was reviewing data from a previous randomized double-blind placebo-controlled trial (RCT) of a bisphosphonate, pamidronate, in blocking bone resorption following the well-known burn-induced systemic inflammatory response [17]. The data obtained had clearly indicated that anti-resorptive agents were safe and effective in preventing bone loss following severe burns in children [17]. When we identified one-third of our RCT patients in a database of pediatric burn patients who completed stable isotope studies of muscle protein kinetics during the first 30 days post-burn, we found that those patients who had received the bisphosphonate did not experience muscle wasting compared to those subjects who had received a placebo, and in contrast to the expected negative muscle protein balance normally seen with burns, these children were in positive muscle protein balance [18]. With both Waning et al. [10] and our group presenting our results at the same meeting in China in 2014, we speculated that the burn patients were being affected by a mechanism similar to what was seen in the mice and patients with metastatic cancer. It was not until 2019 that Pin et al. [19] published the results of in vitro studies utilizing murine C2C12 myoblast cultures containing serum from burned subjects who received either bisphosphonate or placebo or from normal unburned pediatric subjects. They reported that myoblast cultures containing serum from burn patients receiving placebo showed diminished myotube size that was rescued by serum in culture from bisphosphonate-treated patients. Further, placebo-treated burn patient serum in myoblast cultures stimulated the ubiquitin ligase catabolic pathway and suppressed phosphorylation of the anabolic Akt/mTor pathway, while the reverse occurred in cultures with bisphosphonate-containing serum from burn patients. When myoblast cultures with placebo-treated serum were cultured with anti-TGF-β 1–3 antibody, the rescue of myotube size was comparable in magnitude to that in cultures with bisphosphonate-containing serum, indicating that TGF-β was responsible for the reduction in myotube size in pediatric burns, and that preventing bone resorption was sufficient to preserve myotube size in culture and to stimulate the anabolic Akt/mTor pathway while suppressing the catabolic ubiquitin ligase pathway. These latter findings are consistent with part of the mechanism of action of TGF-β on muscle reported by Abrigo et al. [7]. Figure 1 also summarizes what is known of the effects of TGF-β on skeletal muscle. 

The findings in pediatric burn patients, while not addressing the generation of reactive oxygen species seen with cancer metastatic to bone, did demonstrate the influences of TGF-β on the muscle catabolic pathways and suppression of phosphorylation of the anabolic pathway. 

Thus, two very distinct groups of patients, mature adults with cancer with or without metastases, and previously healthy children and adolescents with acute, severe burn injury, have exhibited similar, if not identical, mechanisms of muscle wasting that were influenced by the release of TGF-β from bone. This observation suggests that the release of TGF-β from bone during resorption helps bone control muscle mass and strength. There may also be therapeutic implications of this finding so that factors increasing bone resorption, such as inflammation and possibly immobilization, could be counteracted by the use of anti-resorptive agents, reducing the bone release of TGF-β with consequent sparing of muscle mass and strength. 

A potential third disorder in which TGF-β release may be involved was reported by Jude et al. [20], showing that inhibition of TGF-β1 in septic rats using the TGF-β inhibitor LY364947 protected the diaphragm muscle from sepsis-induced weakness and wasting. Additionally, in vitro studies on old and young muscle from mice by Carlson et al. [21] demonstrated that, at least in mice, old muscle produces excess TGF-β1, but not myostatin, and the former induces high levels of TGF-β phosphorylated Smad3 in muscle satellite cells, interfering with the regenerative capacity of these muscle stem cells. This finding suggests that skeletal muscle from old mice may develop sarcopenia as a result of the increased expression of TGF-β. However, it raises the question as to whether bone resorption releases TGF-β as well, and, if so, what is the relationship between bone TGF-β and muscle TGF-β in elderly humans? Does the presence of TGF-β from bone induce muscle to express more TGF-β? A more recent paper by Zhang et al. [22] that identified an intrinsic TGF-β inhibitor in liver, hemojuvelin, which could suppress TGF-β in muscle from both Duchenne muscular dystrophy and from aging mice, is also consistent with increased muscle TGF-β production in aging animals. 

Both cancer, with or without bone metastases, and pediatric burn injury involve the inflammatory response. In burns, there is also a question of whether immobilization-induced bone resorption contributes to TGF-β release and consequent muscle wasting. At present, the answer to this question is unclear. The other condition that could potentially release TGF-β from bone would be the hyper-resorption resulting from hyperparathyroidism, by a mechanism unrelated to either inflammation or immobilization. To date, this mechanism of pathogenesis of muscle wasting is not studied, although severe cases of hyperparathyroidism have been manifested by muscle wasting [23]. 

In contrast, the hyper-resorption that takes place in Paget’s disease of bone, which can be restricted to a single bone, is associated with an inclusion-body myositis and can also be associated with frontotemporal dementia. There have been several reports of this syndrome, one of which, by Kimonis et al. [24], describes brain histopathology that reveals ubiquitin-positive inclusions located intracellularly in neurons, raising the question as to whether neurons within the brain are subjected to similar degenerative processes as muscle. The abnormality in this condition is located in mutations of the valousin-containing protein gene, VCPp97. Another candidate gene, METTLC21, was identified by Huang et al. [25] in genome-wide association studies. The proteins associated with this gene methylate chaperones involved in the etiology of inclusion body myositis with Paget’s. This etiology has not been associated with TGF-β release from bone, although no studies examining this relationship have been reported to date. 

Additionally, studies by O’Neill et al. [26,27] and Bhardwaj et al. [28] indicate that lack of insulin and IGF-1 receptors, as in diabetes, may upregulate the forkhead box O (FoxO) genes 1–3, which result in increased autophagy and contribute to muscle wasting. This is a complicated area, however, in that FoxO 1 and 3 in muscle and bone are also up-regulated by oxidative stress [29], which can result from any number of underlying causes, including TGF-β release from bone [10] and excessive endogenous glucocorticoid production [29]. In addition, undercarboxylated osteocalcin, which is produced in osteoblasts, is known to increase muscle sensitivity to insulin, and to increase muscle glucose uptake [30], and its role in these streptozotocin and triple FoxO knockout studies is also unknown. 

While the studies of streptozotocin muscle wasting [26] and FoxO knockouts are interesting with regard to involvement in muscle wasting, these studies did not examine bone, so the role of bone loss in the upregulation of FoxO genes is not clear in these cases. As referenced above [29], oxidative stress upregulates FoxO 1 and 3 in bone. In addition, increased autophagy does not play a role in the muscle wasting induced by the liberation of TGF-β from bone [19]. That being said, however, the presence of redundant mechanisms of muscle wasting that do not involve bone loss cannot be excluded. 

## 3. Evidence Suggesting That Immobilization Plays a Role in TGF-β-Mediated Muscle Wasting

We do not have evidence implicating bone-released TGF-β’s involvement in immobilization to date. However, a recent study by Gugala et al. [31] provides suggestive information. Studies of a rat model of critical illness myopathy developed by Llano Diez et al. [32] have shown that by pharmacologically immobilizing rats and mechanically ventilating them for 10 days, a model that reproduces the effects of immobilization and the mechanical ventilation of patients in intensive care for COVID-19 respiratory disease, the loss of appendicular skeletal muscle mass and, in particular, myosin, occurs in tight correlation with the loss of trabecular bone, as determined by serial micro computed tomography of the femurs of rats studied from 0–10 days post-immobilization. While immobilization is the main feature of this model, it is difficult to discount a role of inflammation that may occur as a result of mechanical ventilation. This is because mechanical ventilation itself causes an inflammatory response by upregulating the NLRP3 inflammasome [33], resulting in the increased production of IL-1 by the monocytes and macrophages of the innate immune system. Therefore, the model is not entirely “clean”, in a manner similar to burn injury. However, the tightness of the correlation between muscle and bone loss in this experimental setting is suggestive that bone resorption is involved in the pathogenesis of muscle wasting in this model. The definitive experiment would involve the use of bisphosphonates or other anti-resorptive agents to block the bone loss and then evaluate the muscle wasting. However, studies of atrophic muscle fibers in this condition have been reported to express TGF-β ligands [34]. Similarly, studies of sarcopenia have reported an increase in circulating TGF-β1, phosphorylated Smad 3, and myostatin, again suggesting the involvement of TGF-β1 in these conditions [35]. Yet, it is unclear whether muscle myostatin is upregulated by TGF-β released from bone, although, as we have seen [21], this may not occur in aging, sarcopenic muscle. However, this relationship has not been studied in other conditions, so it is not clear that this is categorically the case. 

## 4. Unanswered Questions

While we have suggestive evidence that bone resorption is involved in muscle wasting, several questions need to be addressed before we can know whether anti-resorptive therapy is universally feasible in cases of bone resorption-associated muscle wasting. 

The first question would be whether there is a threshold for the rate of resorption above which TGF-β is released in quantities sufficient to produce muscle wasting. The cases of bone metastases, cancer inflammation, and severe burn injury may produce rates of resorptive bone loss that are sufficient to liberate TGF-β, but what about smaller burns? Children with ≤20% total body surface area burns did not demonstrate reduced bone mineral density [36]. Whether they experience muscle wasting to the same degree as the hypercatabolic patients with greater body surface area injury is not clear. What about hyperparathyroidism? Does the elevation of serum PTH dictate the amount of bone loss and muscle wasting? What about the degree of immobilization or inactivity? None of this is known. 

A second question is whether, during resorption, all minerals and proteins are released in the same proportion. For example, bone contains hormones such as undercarboxylated osteocalcin, which may be anabolic to muscle, as well as TGF-β, which may be catabolic to muscle. How does the bone know which to release? What are the stimuli that determine what bone releases during resorption? Do other tissues play a role in bone actions?

A third question is how do we identify and distinguish between bone-dependent and bone-independent scenarios of muscle wasting? What are the roles of myokines, such as myostatin and irisin? What are the roles of nutrition and depressive mental states? Much has to be determined with regard to the extent that bone mass and function influence the magnitude of skeletal muscle mass and function. 

We conclude with a speculation. In 2017, Hundeshagen et al. [37], from our institution, reported that in children with burns exceeding 30% total body surface area, long-term myocardial damage was detected in follow-up studies a mean of 8 years following the burn injury. The damage included reduced left ventricular ejection fraction, diastolic dysfunction, and reduced oxygen consumption during exercise. Inasmuch as these patients were cared for at the same institution as the patients we studied, and, in the absence of any changes in therapy, presumably also liberated TGF-β during bone resorption, can we ask whether the TGF-β then affected cardiac muscle as it did skeletal muscle, and that prompt administration of a bisphosphonate such as pamidronate or other anti-resorptive therapy could prevent any deleterious effect of bone-liberated TGF-β on cardiac muscle as well as skeletal muscle? Supporting this speculation are data from Khalil et al. [38], demonstrating in mouse experiments that selective deletion of TGF-β receptors 1 and 2, but not Smad 2/3, attenuated the cardiac hypertrophic response to overflow pressure stimulation. Carefully conducted prospective studies should be able to resolve the issue for burns patients as well as other patients suffering from disorders of bone resorption. 

## Data Availability

Not applicable.
